# Failure Rate of Medical Treatment for Miscarriage Correlated with the Difference between Gestational Age According to Last Menstrual Period and Gestational Size Calculated via Ultrasound

**DOI:** 10.3390/jcm12196112

**Published:** 2023-09-22

**Authors:** Ohad Gluck, Elad Barber, Matan Friedman, Ohad Feldstein, Ori Tal, Ehud Grinstein, Yossi Mizrachi, Ram Kerner, Michal Saidian, Mai Menasherof, Ron Sagiv

**Affiliations:** 1 Department of Obstetrics and Gynecology, Wolfson Medical Center, Holon 58100, Israel; ohadgluck@gmail.com (O.G.); elad.barber@gmail.com (E.B.); ohad60@gmail.com (O.F.); orital@rocketmail.com (O.T.); udigrin@gmail.com (E.G.); mizrachi.yossi@gmail.com (Y.M.); ram_kerner@yahoo.com (R.K.); sagivr@wmc.gov.il (R.S.); 2 Sackler Faculty of Medicine, Tel Aviv University, Tel Aviv 6997801, Israel; mich4726@gmail.com (M.S.); maichimena@gmail.com (M.M.)

**Keywords:** miscarriage, misoprostol, medical treatment

## Abstract

**Objective**: To study whether the interval between gestational age calculated using the last menstrual period (GA-LMP) and gestational age calculated via ultrasound (GA-US) is correlated with the success rate of medical treatment in cases of miscarriages. **Methods**: This was a retrospective cohort study conducted in a gynecology unit in a tertiary medical center. Women who underwent medical treatment with Misoprostol for miscarriage at the Edith Wolfson Medical Center between 07/2015 and 12/2020 were included. Incomplete or septic miscarriages, multiple pregnancies, patients with irregular periods, and cases of missing data were excluded. Failure of medical treatment was defined as the need for surgical intervention due to a retained gestational sac, severe bleeding or retained products of conception. The cohort study was divided into two groups: patients with successful treatment and patients for whom surgical intervention was eventually needed. We performed both a univariate and multivariate analysis in order to identify whether a correlation between GA-LMP and GA-US interval is indeed a factor in the success rate of a medical abortion. **Results**: Overall, 778 patients were included in the study. From this cohort 582 (74.9%) had undergone a successful medical treatment, while 196 (25.1%) required surgical intervention due to the failure of medical treatment, as defined above. The GA-LMP to GA-US interval (in weeks) was 2.6 ± 1.4 in the success group, while the GA in the failure group was 3.1 ± 1.6 (*p* < 0.001). After performing a multivariant regression analysis, we were able to show that the GA-LMP to GA-US interval was found to be independently correlated with an increase in the treatment failure rate (aOR = 1.24, CI 95% (1.01–1.51), *p* = 0.03). **Conclusions**: In cases of miscarriage, longer GA-LMP to GA-US interval has been shown to be an independently correlated factor to lower success rate of the medical treatment option.

## 1. Introduction

The term “miscarriage” encompasses a range of early pregnancy complications, necessitating clear and standardized terminology. Prior to 2005, various definitions were in use across different countries and societies, resulting in confusion and hindrances in research and medical practices. In response, the European Society of Human Reproduction and Embryology (ESHRE) took a significant step by introducing revised definitions for early pregnancy events in 2005 and 2017 [1].

Under these revised definitions, a “biochemical loss” occurs after a positive urinary human chorionic gonadotropin (hCG) test or elevated serum β-hCG levels but before ultrasound or histological confirmation. These losses generally occur before 6 weeks of gestation. In contrast, “clinical miscarriage” and “missed miscarriage” are confirmed by ultrasound or histological evidence, typically before 22 completed weeks of gestational age, when the embryo or fetus is nonviable and not spontaneously expelled from the uterus.

These definitions further categorize “clinical miscarriage” into “early clinical pregnancy losses” (before 12–14 gestational weeks) and “late clinical pregnancy losses” (between 12–14 and 21 weeks), aiding healthcare professionals in providing precise care.

Despite significant advancements in reproductive medicine, clinical miscarriages remain common. Research suggests a rate of early pregnancy loss between 10% and 30% are influenced by factors like maternal age, parity, medical history, and others.

Missed miscarriage accounts for approximately 10–30% of clinically diagnosed pregnancies [2] and is defined as intrauterine fetal death without expulsion of pregnancy products, with multiple factors influencing this rate [2], such as maternal age, parity, gravity, past medical and obstetrical history, and other factors can contribute to the likelihood of experiencing miscarriage symptoms [3].

In recent years, advancements in related sub-fields have led to a better understanding of the underlying causes of miscarriages, improved prevention strategies, and more effective treatments [4].

Surgical evacuation, once widely performed, boasts a 95% [5] success rate but raises concerns about costs and potential complications. Besides short-term complications, such as infection and bleeding, future infertility caused by intrauterine synechia may be unacceptable for women with miscarriages who have not yet fulfilled their motherhood desires. According to the literature, expectant or medical management might be more suitable instead of surgical evacuation [5].

Expectant management, while an alternative, carries risks and uncertainties with unpredictable success rates ranging from 25 to 76% [6]. Waiting for spontaneous expulsion of uterine content also referred to as expected management might cause too much time, during which women may suffer uncertainty and anxiety [6]. More important, in many cases additional surgical intervention is warranted owing to failure, a result that might cause emotional distress [6]. Therefore, expectant management is not recommended for early miscarriage due to the risks of emergency surgical treatment and blood transfusion [6].

The most common medical treatment for first trimester miscarriages is misoprostol [7]. Administered vaginally, it offers success rates ranging from 70% to 90% [8,9,10,11]. Success is defined as medical treatment without the requirement of additional surgical treatment, though up to 30% may require subsequent surgical intervention [12]. In the recent Cochrane review, all forms of treatment were shown to have better results then expected management, with D&C and Misoptostol and Mifepristone showing the best results.

Misoprostol is a synthetic prostaglandin analog that was originally used to treat duodenal ulcers [4]. In the world of gynecology, it is used for cervical ripening before surgical procedures and for the treatment of miscarriage [7]. No major side-effects have been reported previously, and it is considered safe [13]. Vaginal administration of misoprostol is usually associated with less gastrointestinal side effects and abdominal pain as compared to oral administration [13]. 

However, to optimize the effectiveness of Misoprostol treatment, it is imperative to identify factors that can potentially predict treatment failure. Prior research has highlighted certain variables that could play a role, such as assisted reproductive treatment (ART), primigravidity, and vaginal bleeding before administration of the medication [14,15,16]. Machtinger et al. [14], in their work, have identified women with low gravity as having higher chances for success in medical treatment based on misoprostol alone; Creinin et al. [16] have also noted parity as a key factor in the success of the medical treatment, but also noted active vaginal bleeding.

In recent years, a new treatment arose, the combination of Mifepristone–Misoprostol regime, which was shown to be superior to the Misoprostol only regime [17].

Another critical aspect worth considering in cases of miscarriage is the potential discrepancy between gestational age as determined by the last menstrual period (GA-LMP) and the gestational age calculated via ultrasound measurement (GA-US). While GA-LMP represents the duration since the first day of the last menstrual cycle until the miscarriage diagnosis (applicable to patients with regular menstruations), GA-US denotes the specific time and phase when gestational tissue ceased development.

This disparity in gestational age measurements implies that there might be a period during which non-developing gestational tissue remains within the uterus. This time interval between GA-LMP and GA-US could lead to the adherence of gestational tissue to the uterine walls, potentially hindering successful uterine expulsion and subsequently reducing the chances of Misoprostol treatment success.

As such, we aimed to conduct an in-depth investigation into whether the interval between GA-LMP and GA-US correlates with the failure rates of Misoprostol treatment in cases of miscarriage. By identifying this potential correlation, we hope to shed light on possible mechanisms underlying treatment resistance and pave the way for improved management strategies. Understanding these factors can help healthcare providers make informed decisions, enhance patient outcomes, and ensure that women experiencing miscarriages receive the most appropriate and effective care.

## 2. Materials and Methods

We conducted a retrospective cohort study at a single university-affiliated tertiary medical center to investigate the efficacy of Misoprostol in managing first-trimester missed miscarriage (MA) in women. This study, approved by the Institutional Review Board (Registry No. 0004-20-WOMC), focused on medical files of all women who received Misoprostol treatment between July 2015 and December 2020 for first-trimester MA.

At the beginning of the year 2021, as new data arose regarding the superiority of the mifepristone–misoprostol regimen over the misoprostol only regimen, our departmental protocol was amended accordingly. Therefore, the study period included only patients who were treated using the misoprostol only regimen. 

Inclusion criteria were either anembryonic gestation or embryonic death, as long as pregnancy age, as measured by transvaginal sonography (TVS), correlated with 12 weeks gestation or less. However, patients were excluded if they chose expected management, had incomplete or septic miscarriage, multiple pregnancies, or if their cases had irregular periods, missing data, or were lost to follow-up. The diagnosis of early pregnancy loss was meticulously assessed following the guidelines published by the Society of Radiologists in Ultrasound Multispecialty Panel on Early First Trimester Diagnosis of Miscarriage and Exclusion of a Viable Intrauterine Pregnancy [18].

The medical management consists of the administration of 4 tablets of 200 µg of misoprostol (Cytotec, Piramal Healthcare Ltd., Morpeth, UK) vaginally, accumulating to the total dosage of 800 µg on Day 1—the four tablets are placed in the posterior fornix of the vagina by the attending physician. Patients were closely monitored for 30 min and then discharged with a follow-up visit scheduled for Day 4. If complete expulsion was not achieved by Day 4, a second dose of 800 µg of misoprostol was administered vaginally, and another follow-up visit was scheduled for Day 7. In cases where complete expulsion was still not achieved, elective D&C was considered, and emergency D&C was performed in cases of severe bleeding.

Complete expulsion was defined as the absence of a gestational sac and an endometrial thickness of ≤15 mm per TVS.

Furthermore, all patients were advised to undergo an assessment by their gynecologists immediately following their subsequent menstrual period and to seek assistance from our unit if retained products of conception (RPC) were suspected, which were subsequently treated through operative hysteroscopy.

To determine the gestational age, we calculated the GA-LMP based on the patient’s reported last menstrual period (LMP) for those with a regular menstrual cycle. In cases of in vitro fertilization (IVF), GA-LMP was calculated according to the date of embryo transfer. Additionally, GA-US was determined through a TVS examination. Both were calculated at the time of the diagnosis.

### Statistics

The primary outcome was treatment failure, defined as any need for surgical intervention (elective or emergency D&C or operative hysteroscopy).

The cohort was divided according to the success or failure of medical treatment, as previously defined. A univariate analysis was used to compare the groups. 

Data analysis was performed with Epi info 7 (Centers for Disease Control and Prevention, Atlanta, GA, USA). Continuous parameters were compared using an Anova test or Kruskal–Wallis, and categorical variables using a chi-square or Fisher exact test, as appropriate. A *p* value of < 0.05 was considered statistically significant.

A multivariate logistic regression analysis model was used to identify the independent associations with treatment failure, which served as the dependent variable. The following confounders served as independent variables: GA-LMP and GS-US interval, maternal age, maternal body mass index (BMI), parity, US finding at diagnosis, and ART.

## 3. Results

**Figure 1:**
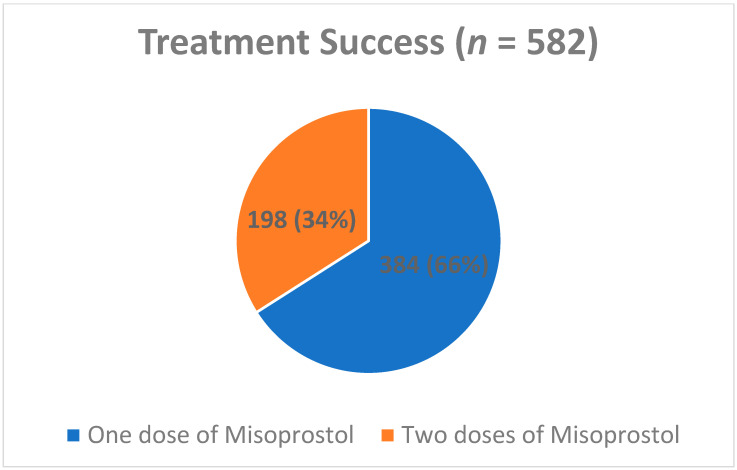
Treatment success cases. Data are presented as n (%) showing the treatment protocol success rate.

**Figure 2:**
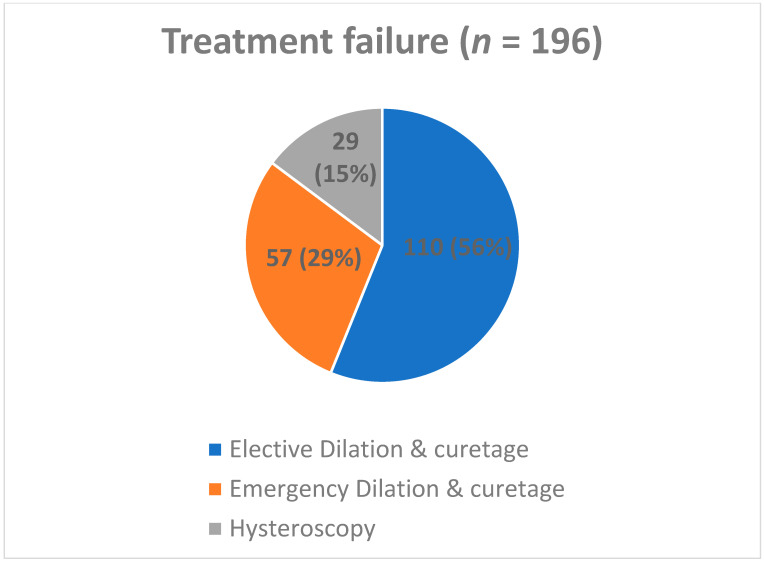
Treatment following failure. Data are presented as n (%) showing the treatment provided in case of failure of medical treatment.

**Figure 3:**
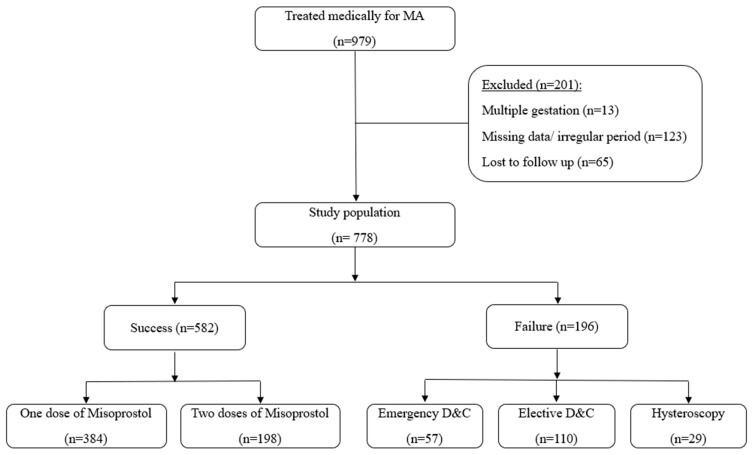
Study population.

Overall, 582 (74.8%) patients were included in the success group (Figure 1), and 196 (25.2%) in the failure group (Figure 2). Of the patients in the success group, 384 (66.2%) completed treatment after a single dose of Misoprostol, while 198 (33.8%) needed a second dose. Of the patients in the failure group, 110 (56.1%) underwent an elective D&C, 57 (29.1%) needed an emergency D&C, and 29 (14.8%) underwent operative hysteroscopy (Figure 3). 

There were no differences between the groups in baseline characteristics: Maternal age was 32.7 ± 6.3 years in the success group and 33.2 ± 5.6 years in the failure group, *p* = 0.5. BMI was 22.9 ± 4.3 kg/m^2^ in the success group vs. 23.3 ± 4.5 kg/m^2^ in the failure group, *p* = 0.6. There was also no difference in obesity (2.4% in the success group and 2% in the failure group, *p* = 0.5) and smoking (6.4% in the success group and 7.7% in the failure group, *p* = 0.5) rates between the two. No differences were identified in the obstetrical history between the groups: mean gravidity was 3.0 ± 1.9 for the success group and 3.0 ± 1.7 for the failure group, *p* = 0.8, mean parity was 1.3 ± 1.3 for the success group and 1.4 ± 1.3 for the failure group, *p* = 0.1; The rate of prior miscarriage was 37.2% in the success groups and 40.8% in the failure group, *p* = 0.8, and usage of Assisted reproductive technology was 43.7% in the success group and 38.4% in the failure group (Table 1).

There were no differences between the groups regarding the US findings at diagnosis: the rates of anembryonic and embryonic pregnancy were 32.7% and 67.3% in the success group, respectively, and 28.1% and 71.9% in the failure group, respectively, *p* = 0.5. The GA-LMP was lower in the success group compared to the failure group (8.6 ± 1.5 vs. 9.6 ± 1.6 weeks, respectively; *p* < 0.001). However, GA-US was similar between the groups (6.4 ± 1.1 for the success group and 6.6 ± 1.4. for the failure group, *p* = 0.2). The LMP-US interval (in weeks) was 2.6 ± 1.4 in the success group and 3.1 ± 1.6 in the failure group (*p* < 0.001) (Table 2). 

Information as to previous D&C procedures were not included as previous studies regarding the success rate did not show any impact of the number of D&C underwent by the patient.

Based on multivariant regression analysis, we found that LMP-US difference was directly correlated with the risk for treatment failure (OR = 1.24 95% CI (1.01–1.51), *p* = 0.03) (Table 3). No other factors were found to be associated with the risk for treatment failure, including maternal age (OR = 0.99 95% CI (0.94–1.04), *p* = 0.8), maternal BMI (OR = 0.98 95% CI (0.92–1.04), *p* = 0.62), prior deliveries (OR = 1.01 95% CI (0.75–1.34), *p* = 0.94), embryonic pregnancy (OR = 1.3 95% CI (0.86–1.97), *p* = 0.21), and ART (OR = 0.71 95% CI (0.4–1.51), *p* = 0.24).

## 4. Discussion

### 4.1. Main Findings

In our study, we found a correlation between GA-LMP (Gestational Age at Last Menstrual Period) and GA-US (Gestational Age determined by Ultrasound) interval and treatment failure. Our findings indicate a strong correlation between the magnitude of the GA-LMP to GA-US difference and the likelihood of treatment failure. This critical insight sheds light on an important aspect of reproductive health and treatment efficacy.

Expanding on our results, we have also observed a noteworthy trend, although not yet statistically proven, which provides additional support to the well-established notion that higher maternal age and Body Mass Index (BMI) are potential factors contributing to an increased likelihood of treatment failure. While we acknowledge that statistical significance has not been established at this stage, the consistency of this trend in our data suggests its potential importance in future research. 

### 4.2. Strengths and Limitations

Our study has a few strengths. First, to the best of our knowledge, this is the first study to investigate the correlation between the GA-LMP to GA-US interval to the rates of treatment failure. Second, data were collected in a single tertiary center, in which treatment was conducted under one protocol, and the decision to intervene surgically was made using strict criteria. Third, as far as we know, this is the largest study conducted to date regarding predictors of medical treatment success and failure of MAs.

Our study is not free of limitations. To begin with, we did not analyze long-term consequences, such as the amount of bleeding, and time to future fertility. Moreover, patients who were treated for MAs at our institute may have been diagnosed and treated for RPC in neighboring hospitals and hence lost to follow-up. Nevertheless, the rate of follow-up loss among the “success” group was less than 10%, and given the rate of 25% for treatment failure among our study population, it is safe to assume that our study’s results would not be affected significantly. Lastly, it is possible that US measurements at time of miscarriage diagnosis might not reflect the exact GA when pregnancy stopped developing, as the gestational sac may shrink after the development stopped.

Adding to the above, it is also worth noting that another limitation is due to the retrospective nature of the research, leading to collection bias.

### 4.3. Interpretation

The overall success rate in our study was 74.8%, which is similar to rates reported in the literature [10,14,19,20].

A number of factors have been shown to be associated with the success of medical treatment for MAs. Creinin et al. [21] conducted a retrospective study of 485 patients and reported that Misoprostol is highly successful in patients present with active bleeding and nulliparous women. Stockheim et al. [22] retrospectively studied 220 patients and found that medical treatment is especially successful among women who conceived after ART and patients with only one or two previous pregnancies. In our study, however, we did not find any association between parity or ART with failure rate. Banerjee et al. [20] analyzed 52 cases of MAs and found that the mifepristone–misoprostol regimen is less effective when serum progesterone is lower than 10 nmol/L. This study is especially interesting in the context of our study, since its findings may be associated with the theory we based our study upon. We hypothesized that the longer the non-developing (or non-viable) gestational tissue remains in the uterus, the higher the chances for local adherence of the remaining non-viable tissue to the uterine walls, thus the higher the risk for the failure of both expected management as well as the medical treatment. It is reasonable to assume that low progesterone levels result from the physiological state described above—a gestational tissue which is subject to degradation. Therefore, it is not surprising that both low progesterone levels and a high GA-LMP to GA-US difference are associated with treatment failure [22].

The combined mifepristone–misoprostol regimen has become the preferred treatment for EPL (Early Pregnancy Loss) of many practitioners, as it was shown to be superior to the misoprostol-only regimen. Several studies conducted over time have shown greater success rate in passing the gestational sac and lower rates in the need for a surgical intervention to complete the miscarriage [8,23,24,25,26,27,28,29]. It is worth noting that no difference was found in the adverse events between the two groups.

However, for regulatory reasons, the misoprostol-only regimen is the most common regimen used in Israel, and it is also the recommended regimen in many international guidelines and has also been proven in several works, including that of Mizrachi et al. [10]. There is a paucity of literature describing prediction factors for mifepristone–misoprostol treatment failure [14]. Therefore, we believe that the present study’s outcome, which is in line with other misoprostol-only studies, carries worldwide relevance and clinical significance.

Another field that requires further investigation is the field of misoprostol treatment and more specifically the interval and dosage of misoprostol [10,13,19,24,25]. Research carried out in this area has shown both option of 600 µg and 800 µg of misoprostol as an effective and safe treatment of the termination of missed miscarriage, both in the success rate, the effect of long term reproductive outcome, etc. 

## 5. Conclusions

We have shown that the difference between GA-LMP to GA-US is directly corelated to treatment failure. This data should be taken into account when counseling patients regarding the optimal treatment for Missed miscarriage.

## Figures and Tables

**Table 1:** Baseline characteristics of the study groups.

	Failure (196)	Success (582)	*p* Value
Maternal age (years)	33.2 ± 5.6	32.7 ± 6.3	0.5
BMI (kg/m^2^)	23.3 ± 4.5	22.9 ± 4.3	0.6
Obese (BMI > 30 kg/m^2^)	4 (2.0)	14 (2.4)	0.5
Smoking	15 (7.7)	37 (6.4)	0.5
Gravidity (mean)	3.0 ± 1.7	3.0 ± 1.9	0.8
1	39 (19.9)	152 (26.1)	0.4
2–4	126 (64.3)	319 (54.8)	0.4
≤5	31 (15.8)	111 (19.1)	0.4
Parity (mean)	1.4 ± 1.3	1.3 ± 1.3	0.1
0	54 (27.6)	210 (36.1)	0.3
1–2	109 (55.6)	280 (48.1)	0.3
≤3	33 (16.8)	92 (15.8)	0.3
Prior miscarrige (yes)	238 (40.8)	73 (37.2)	0.8
Assisted reproductive technology	58 (38.4)	196 (43.7)	0.3

Data are presented as n (%) or mean ± standard deviation. BMI—Body mass index.

**Table 2:** Baseline pregnancy characteristics of the study groups.

**US Findings at Diagnosis**			
	Failure (196)	Success (582)	*p* Value
Anembryonic pregnancy	55 (28.1)	190 (32.7)	0.5
Embryonic pregnancy	141 (71.9)	392 (67.3)	0.5
**Gestational Age at Diagnosis**			
GA-LMP	9.6 ± 1.6	8.6 ± 1.5	<0.001
GA-US	6.6 ± 1.4	6.4 ± 1.1	0.2
GA-LMP to GA-US interval	3.1 ± 1.6	2.6 ± 1.4	<0.001

Data are presented as n (%) or mean ± standard deviation. GA-LMP—Gestational age calculated via Last menstrual period, GA-US—Gestational age calculated via ultrasound.

**Table 3:** Logistic regression model for medical treatment failure.

	OR	95% C.I		*p*
		Lower	Upper	
LMP-US interval (weeks)	1.24	1.01	1.51	0.03
Maternal age (years)	0.99	0.94	1.04	0.80
Maternal BMI (kg/m^2^)	0.98	0.92	1.04	0.62
Prior deliveries	1.01	0.75	1.34	0.94
Embryonic pregnancy	1.3	0.86	1.97	0.21
Assisted reproductive technology	0.71	0.40	1.51	0.24

OR: odds ratio; C.I: confidence interval; LMP—Last menstrual period; US—ultrasound; BMI—Body mass index.

## Data Availability

Not applicable.

## Abbreviations

GA-LMP	Gestational age calculated by last menstrual period
GA-US	Gestational age calculated by ultrasound
D&C	Dilation and Curettage
EPL	Early Pregnancy loss
TVS	Trans Vaginal ultrasound
RPC	Retained Product of Conception
IVF	In Vitro Fertilization
